# Evaluation of Femoropopliteal In-Stent Restenosis Characteristics Stratified by Stent Design

**DOI:** 10.3390/jcm12237225

**Published:** 2023-11-22

**Authors:** Elias Noory, Tanja Böhme, Jonas Salm, Ulrich Beschorner, Larissa Endress, Roaa Bollenbacher, Dirk Westermann, Thomas Zeller

**Affiliations:** 1 Department of Cardiology and Angiology, University Heart Centre Campus Bad Krozingen, Faculty of Medicine, University of Freiburg, 79189 Bad Krozingen, Germany; 2 Medical Faculty, Albert-Ludwigs-University Freiburg, 79110 Freiburg im Breisgau, Germany

**Keywords:** peripheral artery disease, femoropopliteal artery, endovascular therapy, stent, in-stent restenosis

## Abstract

Purpose: To evaluate the potential differences in characteristics of femoropopliteal in-stent restenosis (ISR) stratified by stent design with a focus on the swirling flow-inducing BioMimics 3D helical centerline stent. Methods: Patients with ISR of the superficial femoral and popliteal arteries undergoing reintervention were included in this study. The primary endpoint was the angiographic localization and extent of restenosis or reocclusion with the following five different stent systems: SMART Control stent, Supera peripheral stent, GORE^®^ VIABAHN^®^ endoprosthesis, BioMimics 3D stent, and Zilver^®^ PTX^®^ stent. Results: 414 ISR lesions were analyzed, affecting 236 Supera stents, 67 BioMimics 3D stents, 48 Zilver^®^ PTX^®^ stents, 38 SMART Control stents, and 25 VIABAHN^®^ endoprostheses. The mean stent diameter and length were 5.7 ± 0.77 mm and 121.4 ± 94.8 mm, respectively. ISR included 310 (74.9%) lesions with 1 stent, 89 (21.5%) lesions with 2 stents, 14 (3.4%) lesions with 3 stents, and 1 lesion (0.2%) with 4 stents. Most lesions presented as reocclusions (67.4%) rather than focal (13.3%) or diffuse restenoses (19.3%). No significant differences in ISR lesion morphology were found. By trend, BioMimics 3D stent lesion extension was more focal (16.4% versus 12.7%, *p* = 0.258), with the highest proportion of lesions in which only the proximal stent third was affected (9.0% versus 5.8%, *p* = 0.230), as compared to the average of the other four devices. The occlusion rate was the second lowest for the BioMimics 3D stent (64.2 vs. 68.0%, *p* = 0.316). Risk factors for restenosis or occlusion were active smoking, pre-interventional occlusion, and popliteal intervention. Conclusion: Our results suggest that the helical centerline stent design of the BioMimics 3D stent, which results in a swirling flow with increased wall shear stress, may offer protective properties over straight stent designs, including DES and endoprosthesis, regarding localization and extension of restenosis. Prospective, randomized studies are warranted.

## 1. Introduction

A major challenge arising from the endovascular treatment of femoropopliteal lesions using stents is the development and therapy of in-stent restenosis (ISR) [1,2].

Several factors play a role in the development of ISR, one of which is blood flow pattern. Despite the impact of blood flow patterns on vascular disease development, including stenosis and enlargement, there are few studies on the impact of blood flow patterns on restenosis development [3].

Stent geometry inevitably interferes with blood flow and, consequently, has a significant effect on hemodynamics [4].

Furthermore, the surface of the stent plays a crucial role. Blood flow stagnation at specific sites promotes the accumulation of atherogenic lipids and platelet and granulocyte adhesion to the vessel wall, resulting in the increased formation of atherosclerotic plaques and intimal hyperplasia [5].

One approach to overcome this limitation in stent implantation is the development of a 3D helical centerline stent system that creates a swirling flow, a controlled spiral flow of blood [6], suppressing or reducing the extent of undesired flow disturbances and thereby potentially preventing the development of restenosis [5].

An additional effect is damage reduction to the vessel wall caused by lateral forces [7].

The aim of this work is to evaluate the potential differences in the angiographic characteristics of femoropopliteal ISR based on stent design, with an emphasis on the impact of the 3D helical centerline stent geometry.

## 2. Methods

### 2.1. Patient Population

This retrospective study included patients treated for femoropopliteal ISR from June 2015 to December 2020, selected from a prospectively maintained database. The Ethics Committee of Albert-Ludwigs-University Freiburg provided study approval (20-1254) on 19 November 2020. The inclusion criterion was the presence of ISR after the implantation of one of the five stents listed below.

**Figure 1:**
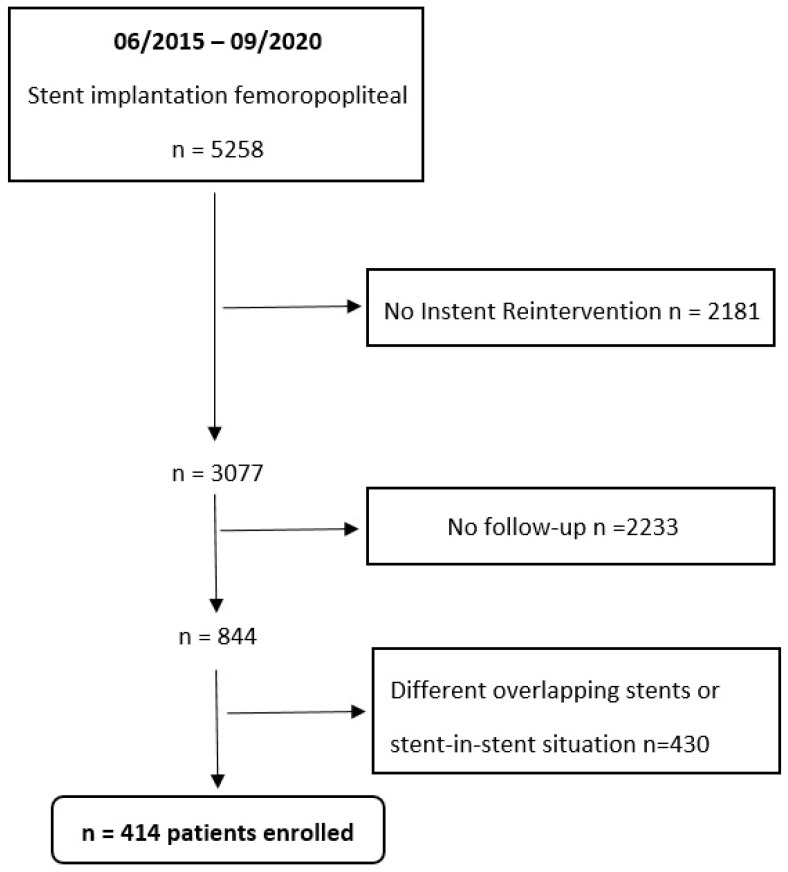
Study flow chart.

Patients with untreated iliac artery inflow disease, as well as femoropopliteal lesions treated with different overlapping stent types, were excluded. Patients who were treated for stent-in-stent restenosis were also not included. Figure 1 shows a flow chart for the study. Table 1 shows baseline characteristics.

### 2.2. Included Stent Designs

**Figure 2:**
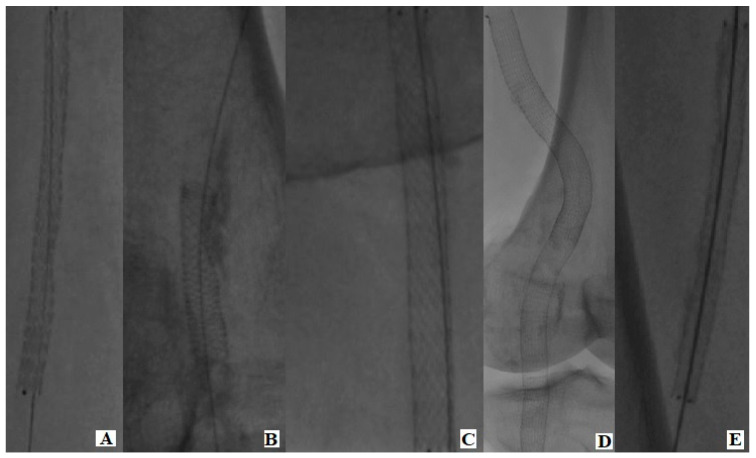
Fluoroscopy of stent designs in use. (**A**) BioMimics 3D stent, Veryan Medical Ltd., Galway, Ireland; (**B**) Supera peripheral stent, Abbott Vascular Corporation, Santa Clara, CA, USA; (**C**) Zilver^®^ PTX^®^, Cook Medical, Bloomington, IN, USA; (**D**) GORE^®^ VIABAHN^®^ endoprosthesis, Gore & Associates Inc., Flagstaff, AZ, USA; (**E**) SMART Control stent, Cordis Endovascular, Fremont, CA, USA.

Five stent brands and designs were selected as follows: a first-generation laser-cut slotted tube stent (SMART Control stent, Cordis Endovascular, Fremont, CA, USA), a wire-interwoven stent (Supera peripheral stent, Abbott Vascular Corporation, Santa Clara, CA, USA), a PTFE-covered endoprosthesis (GORE^®^ VIABAHN^®^ endoprosthesis, Gore & Associates Inc., Flagstaff, AZ, USA), a 3D helical centerline stent (BioMimics 3D stent, Veryan Medical Ltd, Galway, Ireland), and a polymer-free paclitaxel-coated stent (Zilver^®^ PTX^®^, Cook Medical, Bloomington, IN, USA) (Figure 2).

Table 2 summarizes the lesion characteristics of the index intervention, i.e., of the initial stent implantation. The BioMimics 3D stent and Supera stent were predominantly used in the distal superficial femoral artery (SFA) and in the popliteal artery. Two hundred seventy-seven of the index lesions were chronic total occlusions (66.9%). The mean stent diameter was 5.7 ± 0.77 mm, and the mean stent length was 121.4 ± 94.8 mm. In 310 lesions (74.9%), only 1 stent was implanted, and in 89 (21.5%), 2 were implanted. A maximum of four stents were used (n = 1, 0.2%).

### 2.3. Study Endpoints

The primary endpoint of this study was the angiographic characterization of localization and the extent of restenosis or reocclusion within the different femoropopliteal stent systems. For this purpose, ISRs were classified according to the Tosaka classification [8]. Here, stenoses and occlusions are visually assessed by angiographic imaging and grouped into three classes: focal, diffuse, and occluded. A focal stenosis is present if the narrowing is limited to a short section (≤50 mm in length). Diffuse stenosis is defined as narrowing over more than 50 mm in length. In addition, the localization was described as follows: edge stenosis, proximal, middle, and distal third of the stent segment.

The secondary endpoints were time from implantation to restenosis (defined as stenosis with a peak systolic velocity ratio of >2.5, measured on a color-flow duplex ultrasound), independent predictors for restenosis or stent occlusion, and clinical stage (Rutherford Becker class), as assessed at the time of reintervention [9].

### 2.4. Statistical Analysis

All study-relevant information was analyzed in the statistical program IBM SPSS Statistics (version 25; IBM Corp., Armonk, New York, NY, USA). The statistical significance was set at *p* < 0.05.

Categorical variables are expressed as frequencies and percentages. Continuous variables are expressed as mean values with standard deviations.

To evaluate differences between the groups, a one-factor analysis of variance (ANOVA) and a chi-square test were performed.

Freedom from restenosis was evaluated using Kaplan–Meier analysis and compared with the Mantel–Cox log-rank test.

For identification of risk factors for restenosis or reocclusion, we used binary logistic regression analysis by means of a stepwise forward variable selection procedure. The following patient characteristics and treatment details were included in the analysis: smoking status, diabetes mellitus, renal disease, intervention details, and stent location. The results of the regression analysis are given as an odds ratio with 95% confidence intervals. 

## 3. Results

The cohort studied included 414 patients with 236 Supera stents, 67 BioMimics 3D stents, 48 Zilver^®^ PTX^®^ stents, 38 SMART Control stents, and 25 VIABAHN^®^ endoprosthesis lesions. 

**Figure 3:**
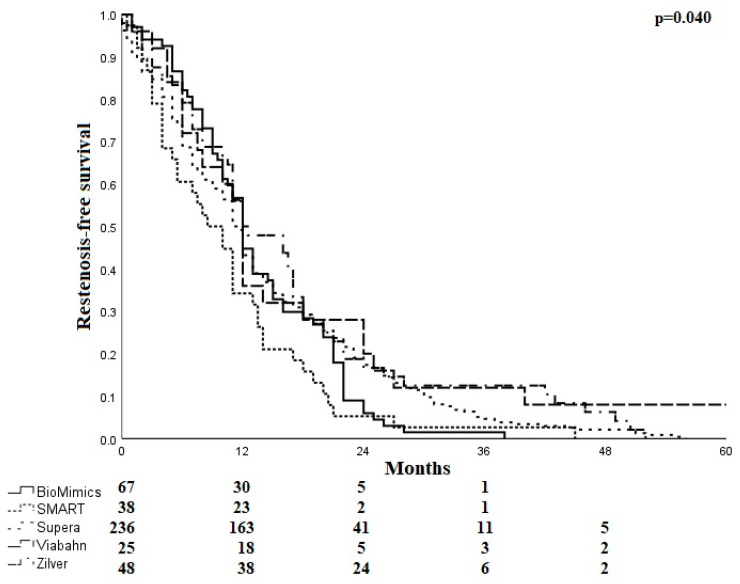
Kaplan–Meier curve for restenosis-free survival.

After stent implantation, the mean time to restenosis ranged from 10.5 ± 8.7 months for SMART stents to 18.9 ± 22.4 months for Viabahn endoprostheses. The mean time to restenosis was 13.3 ± 7.6 months for the BioMimics 3D stent, 14.1 ± 11.6 months for the Supera stent, and 16.6 ± 13.5 months for the Zilver PTX stent. Freedom from restenosis is shown in Figure 3.

The number of ISR occlusions was higher if the index lesion was a CTO, as compared to stenosis (79.4% vs. 43.1%, *p* < 0.001).

Most ISR lesions presented as occlusions (n = 279, 67.4%) rather than focal (n = 55, 13.3%) or diffuse restenoses (n = 80, 19.3%). The proportion of occlusions ranged from 56.3% in Zilver to 76.0% in Viabahn (*p* = 0.354). For focal stenoses, the range extended from 9.7% in Supera to 24.0% in Viabahn (*p* = 0.137). Diffuse stenoses occurred from 10.5% in SMART to 27.1% in the Zilver groups (*p* = 0.036). Viabahn endoprostheses had either focal stenosis or occlusion but no diffuse stenoses. Edge stenosis was present in approximately 20.0%, ranging from 14.6% in Zilver PTX to 24.0% in the Viabahn groups (*p* = 0.553). 

Regarding lesion extension (Table 3), the BioMimics 3D performed slightly differently compared with the other stent systems, with fewer patients affected by a lesion in all thirds (76.1% versus 82.1%, *p* = 0.163). 

In addition, the proportion of patients in which only the proximal third of the stent was affected was 9.0% in the BioMimics stent cohort compared to 5.8% in the other devices (*p* = 0.230). In one case in the BioMimics 3D stent cohort, the stenosis was localized in the middle (1.5%), and in another case, it was localized in the distal segment (1.5%, 0.683). In comparison, in the other stents, the stenosis was in the middle segment in six cases (1.7%) and in the distal segment in seven cases (2.0%, *p* = 0.619).

Stents involving the popliteal segment were more frequently affected by occlusions (73.2%) than in patients without knee involvements (60.8%, *p* = 0.005) (Table 4).

The proportion of occlusions was 67.4% across all stent diameters. The percentage of occlusions was the lowest in the 6 to 6.5 mm stent diameter range (62.1%, *p* < 0.001) (Table 4).

Except for Supera, the longer the total stent length, the more frequently occlusions occurred in all systems, and the shorter the stent length, the more frequently stenoses were seen (Table 4).

Outflow conditions had no significant impact on the proportion of stent occlusions in the entire study cohort; the percentage of occlusions in the overall cohort was 71.5%, with one patent with a below-the-knee vessel, 64.3% with two, and 69.2% with three. However, in the BioMimics 3D and Viabahn cohorts, the proportion of occlusions decreased with the increasing number of open vessels (Table 4).

The mean Rutherford class at baseline in the cohort with an occlusion was 3.4 ± 0.99 and 3.3 ± 0.89 in the cohort with stenosis. Before reintervention, the mean Rutherford class in the cohort with a stent occlusion was 3.57 ± 0.95, and in the cohort with stenosis, it was 3.23 ± 1.2. Rutherford Becker classes at the time of primary stent implantation and at the time of reintervention are shown in Table 5.

Multivariate logistic regression analysis identified active smoking, pre-interventional occlusion, and popliteal intervention as risk factors for restenosis or reocclusion (Table 6).

## 4. Discussion

In the present study, we compared five self-expanding nitinol stent geometries and designs regarding their restenosis or reocclusion characteristics. In summary, potentially due to the small numbers, no significant differences in the percentage of stent occlusions or ISR lesion location within the stent were found.

Special attention was given to the BioMimics 3D helical centerline system, which is designed to potentially suppress the development of neo-intima-related restenosis by creating increased swirling flow-induced wall shear stress [5]. Of note, swirling flow inside the BioMimics 3D stent is created 2 to 3 cm distal to the proximal stent edge; the first 2 centimeters of the stent are straight. Therefore, one focus of the analysis was restenosis location within the course of the stent. We expected to find more restenoses in the proximal compared to the mid and distal thirds of the BioMimics 3D stent and a more uniform lesion distribution in the straight stent designs.

The comparison of the restenosis patterns is limited by an uneven distribution of stent location and stent length between the study cohorts. The BioMimics 3D and Supera stents were primarily used in the distal segment of the SFA and the popliteal artery. Compared to more proximal SFA segments, these vessel segments are characterized by more pronounced leg motion, inducing vessel and stent deformation [10]. The helical design of the BioMimics 3D stent can intercept shortening of the femoropopliteal artery during knee flexion. With straight stents, the compression stress is more localized, increasing the risk of stent fracture [11].

The interwoven Supera stent is also designed helically and is particularly resistant to compression forces. It is, therefore, also well suited for use in distal femoral or popliteal segments and in calcified lesions [12]. 

Regarding the localization of restenosis within the stent, the proportion of stents with all three thirds of the stent affected was highest across all systems. If occlusions were present, all thirds of the stent were almost invariably affected. Across all stents, there were also significantly more occlusions than stenoses. By trend, Zilver PTX (56.3%) and BioMimics 3D (64.2%) presented the lowest occlusion rates compared to Supera (69.1%), SMART (71.1%), Viabahn (76.0%), and the overall cohort (67.4%) (*p* = 0.354), respectively. A design-specific finding of the Viabahn endoprosthesis was lesion presentation, either as total occlusion or focal edge stenosis, as neo-intima hyperproliferation within the endoprosthesis is only reported anecdotical, explaining the high proportion of extensive lesions with this particular device. 

The reduction in neo-intimal hyperplasia by high wall shear stress induced by swirling flow in helical stents might explain why lesions were more focal in the BioMimics 3D stent cohort compared with the other systems. As expected by the design of this particular stent, lesions exclusively located in the proximal third were seen as more frequent by trend as compared to the other stent designs. 

In ten healthy pigs, Caro et al. investigated the effect of helical stent geometry compared with straight stent geometry on self-expanding nitinol stents. Digital subtraction angiography demonstrated the presence of swirling flow in the helical stent. A histological examination showed an average 45% reduction in intimal thickness, i.e., significantly reduced neo-intimal hyperplasia in vessels treated with a helical stent compared with those treated with a straight stent. In addition, significantly reduced intimal hyperplasia was observed in the helical stent design due to the altered wall shear stress, especially in the distal region, whereas it was more pronounced proximally and medially. In straight stents, the intimal thickness is distributed inversely [13]. Shinke et al. also noted swirling flow with decreased intimal hyperplasia, attributing this to increased wall shear stress [14].

A small randomized controlled trial found a significantly better patency rate of the BioMimics 3D stent compared with the straight control stent after two years, indicating the positive influence of swirling flow and the associated increased wall shear stress in helical stents [15]. 

In the present study, the mean time to restenosis was the longest for the Viabahn endoprosthesis, followed by the Zilver PTX stent. Both devices are drug-coated or covered: the Viabahn with PTFE and the Zilver PTX stent with paclitaxel. For the BioMimics, the time to restenosis was shorter but comparable to the Supera stent and longer than after implantation of the SMART stent. 

Stent occlusion occurred more frequently if the index lesion prior to stent implantation was an occlusion, as compared to stenosis (79.4% vs. 43.1%, *p* < 0.001). BioMimics had the lowest occlusion rate if the index lesion was stenosis (17.6%). In multivariate analysis, pre-interventional occlusion was a risk factor for restenosis or reocclusion.

Several studies confirm the link between index lesion occlusion and stent occlusion [8,16,17].

The type of stent patency failure, stenosis vs. occlusion, has significant implications for the treatment success of ISR. Armstrong et al. stratified treatment outcomes after endovascular treatment of femoropopliteal ISR according to Tosaka’s ISR classification. ISR with total occlusion posed a significantly increased risk of recurrence of ISR or occlusion compared with stenotic ISR lesions [16]. In a small retrospective study, Ihnat et al. showed a similar trend comparing the treatment of nitinol stent ISR with PTA regarding restenosis rate, stratified to ISR occlusion vs. stenosis: 67% of stent occlusions required reintervention during follow-up, whereas none of the nine ISR stenoses experienced recurrent ISR or reocclusion over a 17.8-month period [17].

The total occlusion rate in the present study (entire cohort—67.4%, BioMimics cohort—64.2%) is twice as high as reported in previous studies dealing with ISR treatment [8,16]. The majority of the stents in this study were implanted in the distal femoral and extended into the popliteal segment. Popliteal intervention was a predictor of restenosis and reocclusion in multivariate analyses.

Shibuya et al. investigated the relationship between stent location and occlusion. Particular attention was paid to 15 patients who had occlusions in either the common femoral or popliteal artery belonging to study Group A. Group B included patients with occlusions mostly in the SFA. Group A occlusions were more likely to have more severe symptoms than group B. A bypass was also needed more often. The authors hypothesize that the adverse outcomes were caused by the localization of the stent in flexed areas or vessel segments with collaterals [18]. Further studies suggest that stents in the popliteal region have an increased risk of developing stent occlusion due to physiologic vessel motion and associated stent deformities, resulting in intermittent low or no flow and an increased risk of stent thrombosis and stent fractures [19,20,21]. 

The present study showed a clear link between stent length and stent occlusion, with increasing stent length or a chain of stents leading to more occlusions. 

In lesions exceeding 200 mm in length, a chain of overlapping stents was implanted, except in some Viabahn cases, which generally worsens the outcome, presumably because decreasing vessel mobility in overlap zones increases the risk of stent fractures [22]. However, this does not explain the higher occlusion rate of the BioMimics stent in longer lesions compared to the other systems. It is conceivable that the protective effect of swirling flow against neo-intima hyperproliferation decreases over longer distances or is disturbed by the overlap zones of multiple stents. In the BioMimics 3D study, with a mean stented lesion length of 130.8 mm ± 79.2 mm, lesions longer than 150 mm were associated with a significantly lower primary patency rate than shorter lesions [23]. Hong et al. also found significantly higher primary patency in short stents compared with long stents, with long stents extending into the distal popliteal artery or chains of stents being an independent predictor of restenosis [24]. In the multivariate analysis, however, stent length was not a risk factor for restenosis or reocclusion.

The impact of the number of outflow vessels on stent occlusion did vary between the groups. In the multivariate analysis, the number of patent leg vessels was not a risk factor for restenosis or reocclusion. In contrast to the remaining devices, in the BioMimics 3D stent cohort, we saw a relationship between the number of patent outflow vessels and stent occlusions: three patent below-the-knee vessels were associated with a proportion of occlusions of 50.0% vs. 65.5% and 75% in the presence of one or two patent outflow vessels, respectively. The potential protective effect of swirling flow preventing ISR development may be reduced by increased peripheral vascular resistance due to impaired outflow conditions. Some former studies confirm the negative impact of outflow vessel obstruction on stent patency.

London et al. and Hong et al. showed that stent patency is influenced by the number of patent outflow vessels [24,25]. Davies et al. also found that patients who had poor outflow vessel conditions were more often symptomatic, in addition to presenting a higher rate of restenosis [26]. However, Sullivan et al. did not confirm our finding; in their study, the number of patent outflow vessels had no statistically significant effect on the primary patency of the BioMimics 3D stent at 24 months [27,28].

An interesting, not yet widely discussed, question is whether ISR results in different symptoms as compared to the clinical stage before the intervention, in particular if a Viabahn is implanted, potentially covering collateral vessels. In more than half of the patients in our entire cohort, there was no difference in the Rutherford class before the index stent procedure or prior to the reintervention, with the BioMimics group showing the lowest rate of change in the Rutherford class. Worsening of symptoms occurred in 25.9% of the entire cohort, while it was 20.9% for the BioMimics 3D stent cohort. In the Viabahn group, there was a worsening of the symptoms in 32%. However, critical limb ischemia was no more frequent before reintervention (n = 3) than before Viabahn implantation (n = 4). Unfortunately, these data are not comparable to previous studies, as these studies report clinical symptoms at predefined study intervals and included patients who already underwent a TLR during follow-up. Therefore, clinical outcomes such as ankle–brachial index and Rutherford category do not differ significantly in RCTs, as shown in the BioMimics RCT, where improvement of at least one Rutherford category at 12 and 24 months was seen in 86% and 88% of patients with a BioMimics stent, as compared to 82% and 86% with the control stent, respectively [15].

## 5. Limitations

The major limitation is the retrospective descriptive study design, with an uneven number of patients and lesions included in the study sub-cohorts. We cannot rule out that differences in gender, smoking habits, number of stents, stent location, stent diameter, and length, as well as occlusion rates and RBC stage, influenced the results. Due to the small sample sizes in some sub-cohorts, matching patient and lesion characteristics was not possible.

Even if large amounts of patient- and lesion-related data and parameters were collected, there may still be unrecorded factors that may have an impact on ISR development, such as the duration of anti-platelet therapy, combination therapy with oral anticoagulation, the statin dose, and the lipid profile. 

## 6. Conclusions

Our data suggest that the helical centerline stent design of the BioMimics 3D stent, resulting in swirling flow with increased wall shear stress, may offer protective properties and may provide some clinical advantages over straight stent designs, including DES and endoprosthesis, regarding the localization and extension of restenosis.

The findings of this study may provide additional knowledge for further stent design developments and prospective comparative studies.

## Figures and Tables

**Table 1:** Baseline patient characteristics.

	BioMimics	SMART	Supera	Viabahn	Zilver	Total	
	n/%	n/%	n/%	n/%	n/%	n/%	*p*-Value
Total cohort	67	38	236	25	48	414	
Male	33 (49.3)	18 (47.4)	151 (64)	20 (80)	28 (58.3)	250 (60.4)	0.021
Smoker	27 (40.3)	15 (39.5)	64 (27.1)	13 (52)	21 (43.8)	140 (33.8)	0.015
Former smoker	13 (19.4)	13 (34.2)	79 (33.5)	8 (32)	11 (22.9)	124 (30)	0.167
Hypertension	58 (86.6)	31 (81.6)	202 (85.6)	21 (84)	44 (91.7)	356 (86)	0.730
Diabetes mellitus	27 (40.3)	12 (31.6)	86 (36.4)	9 (36)	22 (45.8)	156 (37.7)	0.673
Hyperlipidemia	58 (86.6)	31 (81.6)	208 (88.1)	24 (96)	47 (97.9)	368 (88.9)	0.099
Renal insufficency	18 (26.9)	8 (21.1)	64 (27.1)	5 (20)	12 (25)	107 (25.8)	0.885
Dialysis	3 (4.5)	1 (2.6)	7 (3)	5 (20)	1 (2.1)	12 (2.9)	0.883
Coronary heart disease	21 (31.3)	13 (34.2)	101 (42.8)	10 (40)	24 (50)	169 (40.8)	0.265
Cerebral vascular disease	8 (11.9)	3 (7.9)	44 (18.6)	3 (12)	8 (16.7)	66 (15.9)	0.375
Stroke	8 (11.9)	4 (10.5)	19 (8.1)	1 (4)	8 (16.7)	40 (9.7)	0.308

Values are n (%). A chi-square test was used.

**Table 2:** Lesion and stent characteristics of the index intervention.

	BioMimics	SMART	Supera	Viabahn	Zilver	Total	
	n/%	n/%	n/%	n/%	n/%	n/%	*p*-Value
**Side**							
Left	32 (47.8)	15 (39.5)	112 (47.5)	16 (64)	19 (39.6)	194 (46.9)	0.302
Right	35 (52.2)	23 (60.5)	124 (52.5)	9 (36)	29 (60.4)	220 (53.1)	0.302
**Number of stents**							
1	54 (80.6)		175 (74.2)	10 (40)	38 (79.2)	310 (74.9)	<0.001
2	11 (16.4)	33 (86.8)	51 (21.6)	14 (56)	8 (16.7)	89 (21.5)	<0.001
3	2 (3)	5 (13.2)	9 (3.8)	1 (4)	2 (4.2)	14 (3.4)	0.806
4			1 (0.4)			1 (0.2)	
**Stent localization**							
1	5 (7.5)	15 (39.5)	8 (3.4)		12 (25)	40 (9.7)	<0.001
1,2	7 (10.4)	3 (7.9)	6 (2.5)	1 (4)	4 (8.3)	21 (5.1)	0.063
1,2,3	4 (6)	1 (2.6)	13 (5.5)	9 (36)	8 (16.7)	35 (8.5)	<0.001
1,2,3,4	2 (3)	1 (2.6)	10 (4.2)	12 (48)	1 (2.1)	26 (6.3)	<0.001
1,2,4			1 (0.4)			1 (0.2)	0.944
1,3			1 (0.4)		1 (2.1)	2 (0.5)	0.524
1,3,4			1 (0.4)			1 (0.2)	0.944
1,4			1 (0.4)			1 (0.2)	0.944
2	2 (3)	3 (7.9)	16 (6.8)		5 (10.4)	26 (6.3)	0.334
2,3	5 (7.5)	1 (2.6)	17 (7.2)		3 (6.3)	26 (6.3)	0.552
2,3,4	2 (3)		14 (5.9)	3 (12)		19 (4.6)	0.071
2,4			1 (0.4)			1 (0.2)	0.944
3	9 (13.4)	7 (18.4)	20 (8.5)		8 (16.7)	44 (10.6)	0.062
3,4	16 (23.9)		42 (17.8)		4 (8.3)	62 (15)	0.001
4	15 (22.4)	7 (18.4)	85 (36)		2 (4.2)	109 (26.3)	<0.001
**Mean stent diameter (mm)**	6.2 ± 0.55	6.7 ± 1.1	5.4 ± 0.51	6.0 ± 0.49	6.3 ± 0.45	5.7 ± 0.77	<0.001 *
**Mean stent length (mm)**	117.8 ± 65.7	57.4 ± 54.3	115.1 ± 83.8	311 ± 79.7	108.9 ± 97.3	121.4 ± 94.8	<0.001 *
**Target lesion**							
Stenosis	17 (25.4)	17 (44.7)	80 (33.9)	2 (8)	21 (43.8)	137 (33.1)	0.008
Occlusion	50 (74.6)	21 (55.3)	156 (66.1)	23 (92)	27 (56.3)	277 (66.9)	0.008
**n patent BTK vessels**							
0		2 (5.3)	6 (2.5)		1 (2.1)	9 (2.2)	0.419
1	20 (29.9)	10 (26.3)	76 (32.2)	6 (24)	18 (37.5)	130 (31.4)	0.724
2	29 (43.3)	15 (39.5)	101 (42.8)	7 (28)	16 (33.3)	168 (40.6)	0.495
3	18 (26.9)	11 (28.9)	53 (22.5)	12 (48)	13 (27.1)	107 (25.8)	0.089

**Stent localization**: 1: proximal superficial femoral artery; 2: middle superficial femoral artery; 3: distal superficial femoral artery; 4: popliteal artery; n: number; BTK: below the knee. Values are n (%) or mean ± standard deviation. A chi-square test was used. * ANOVA was used.

**Table 3:** Localization in-stent restenosis.

					95% CI	
System	Stent Third	n	%	SE (%)	LV	UV
BioMimics	1	6	9	3.5	3.8	17.5
	1,2	2	3	2.1	0.6	9.2
	1,2,3	51	76.1	5.2	65	85.1
	1,3	3	4.5	2.5	1.3	11.5
	2	1	1.5	1.5	0.2	6.8
	2,3	3	4.5	2.5	1.3	11.5
	3	1	1.5	1.5	0.2	6.8
SMART	1	2	5.3	3.6	1.1	15.8
	1,2	1	2.6	2.6	0.3	11.6
	1,2,3	30	78.9	6.6	64.2	89.5
	1,3	1	2.6	2.6	0.3	11.6
	2	1	2.6	2.6	0.3	11.6
	2,3	2	5.3	3.6	1.1	15.8
	3	1	2.6	2.6	0.3	11.6
Supera	1	12	5.1	1.4	2.8	8.5
	1,2	6	2.5	1.0	1.1	5.2
	1,2,3	198	83.9	2.4	78.8	88.2
	1,3	4	1.7	0.8	0.6	4.0
	2	4	1.7	0.8	0.6	4.0
	2,3	6	2.5	1.0	1.1	5.2
	3	6	2.5	1.0	1.1	5.2
Viabahn	1	3	12	6.5	3.5	28.7
	1,2	1	4	3.9	0.4	17.2
	1,2,3	20	80	8.0	61.6	91.9
	1,3	1	4	3.9	0.4	17.2
	2	0				
	2,3	0				
	3	0				
Zilver	1	3	6.3	3.5	1.8	15.7
	1,2	2	4.2	2.9	0.9	12.7
	1,2,3	37	77.1	6.1	63.8	87.2
	1,3	2	4.2	2.9	0.9	12.7
	2	1	2.1	2.1	0.2	9.3
	2,3	3	6.3	3.5	1.8	15.7
	3	0				

Standard errors and confidence intervals of localization of stenosis by stent system. CI: confidence interval; n: number; SE: standard error; LV: lower value; UV: upper value; 1: proximal stent third; 2: middle stent third; 3: distal stent third.

**Table 4:** Occlusion or stenosis based on different variables.

	BioMimics	SMART	Supera	Viabahn	Zilver	Total	*p*-Value
	n/%	n/%	n/%	n/%	n/%	n/%	
**Stent localization**	**Occlusion**						
Femoral	18 (56.3)	21 (70)	48 (59.3)	8 (80)	23 (56.1)	118 (60.8)	<0.001
Femoropopliteal	13 (65)	1 (100)	54 (77.1)	11 (73.3)	3 (60)	82 (73.9)	<0.001
Popliteal	12 (80)	5 (71.4)	61 (71.8)	0	1 (50)	79 (72.5)	<0.001
**Stent localization**	**Stenosis**						
Femoral	14 (43.9)	9 (30)	33 (40.7)	2 (20)	18 (43.9)	76 (39.2)	0.004
Femoropopliteal	7 (35)		16 (22.9)	4 (26.7)	2 (40)	29 (26.1)	0.012
Popliteal	3 (20)	2 (28.6)	24 (28.2)		1 (50)	30 (27.5)	0.020
**Stent diameter (mm)**	**Occlusion**						
4	0	0	4 (100)	0	0	4(100)	0.577
5–5.5	5 (100)	0	119 (67.8)	6 (100)	0	130 (69.6)	<0.001
6–6.5	24 (53.3)	14 (63.3)	40 (70.7)	10 (64.7)	20 (57.1)	108 (62.1)	<0.001
7	14 (82.4)	8 (80)	0	3 (100)	7 (53.8)	32 (74.4)	<0.001
8	0	2 (66.7)	0	0	0	2 (66.7)	<0.001
9	0	1 (100)	0	0	0	1 (100)	0.053
10	0	2 (100)	0	0	0	2 (100)	<0.001
**Stent diameter (mm)**	**Stenosis**						
4							
≥5–6			56 (32.2)			56 (30.4)	<0.001
≥6–7	21 (46.7)	8 (36.4)	17 (29.3)	6 (35.3)	15 (42.9)	67 (37.9)	<0.001
7	3 (17.6)	2 (20)			6 (46.2)	11 (25.6)	<0.001
8		1 (33.3)				1 (33.3)	0.023
**Stent lenght (mm)**	**Occlusion**						
≤100	19 (52.8)	24 (68.6)	93 (68.4)	0	16 (51.6)	152 (63.9)	<0.001
>100 to ≤200	17 (73.9)	2 (100)	50 (71.4)	0	5 (55.6)	74 (70.5)	0.001
>200 to ≤300	6 (85.7)	1 (100)	17 (73.9)	9 (69.2)	4 (66.7)	37 (74)	<0.001
>300 to ≤400	1 (100)	0	2 (40)	8 (88.9)	1 (100)	12 (75)	<0.001
>400 to ≤500	0	0	1 (50)	2 (100)	1 (100)	4 (80)	0.008
**Stent lenght (mm)**	**Stenosis**						
≤100	17 (47.2)	11 (31.4)	43 (31.6)		15 (48.4)	86 (36.1)	<0.001
>100 to ≤200	6 (26.1)		20 (28.6)	1 (100)	4 (44.4)	31 (29.5	0.347
>200 to ≤300	1 (14.3)		6 (26.1)	4 (30.8)	2 (33.3)	13 (26)	<0.001
>300 to ≤400			3 (60)	1 (11.1)		4 (25)	0.202
>400 to ≤500			1 (50)			1 (20)	0.931
**BTK n**	**Occlusion**						
0	0	1 (50)	2 (33.3)	0	1 (100)	4 (44.4)	0.571
1	15 (75)	8 (80)	57 (75)	6 (100)	7 (38.9)	93 (71.5)	0.894
2	19 (65.5)	9 (60)	62 (61.4)	6 (85.7)	12 (75)	108 (64.3)	0.793
3	9 (50)	9 (81.8)	42 (79.2)	7 (58.3)	7 (53.8)	74 (69.2)	0.658
**BTK n**	**Stenosis**						
0	0	1 (50)	4 (66.7)	0	0	5 (55.6)	0.478
1	5 (25)	2 (20)	19 (25)	0	11 (61.1)	37 (28.5)	0.042
2	10 (34.5)	6 (40)	39 (38.6)	1 (14.3)	4 (25)	60 (35.7)	0.036
3	9 (50)	2 (18.2)	11 (20.8)	5 (41.7)	6 (46.2)	33 (30.8)	0.002

Femoral: proximal, medial, or distal third of the superficial femoral artery (AFS); femoropopliteal: one or more thirds of the AFS along with the popliteal artery (AP); popliteal: exclusively AP; n: number. Values are n (%). A chi-square test was used.

**Table 5:** Rutherford Becker class at the time of stent implantation and at the time of reintervention.

RBC Index Stent Implantation	BioMimics	SMART	Supera	Viabahn	Zilver	Total	*p*-Value
	n/%	n/%	n/%	n/%	n/%	n/%	
0			1 (0.4)			1 (0.2)	
1			3 (1.3)		1 (2.1)	4 (1)	
2	3 (4.5)		31 (13.1)	6 (24)	6 (12.5)	46 (11.1)	
3	43 (64.2)	20 (52.6)	129 (54.7)	15 (60)	29 (60.4)	236 (57)	
4	10 (14.9)	14 (36.8)	23 (9.7)	3 (12)	5 (10.4)	55 (13.3)	
5	10 814.9)	4 (10.5)	47 (19.9)	1 (4)	7 (14.7)	69 (16.7)	
6	1 (1.5)		2 (0.8)			3 (0.7)	
MV	3.45	3.58	3.35	2.96	3.23	3.35	0.099
RBC in-stent reintervention							
0			1 (0.4)	1 (4)		2 (0.5)	
1	2 (3)		7 (3)	3 (12)	1 (2.1)	13 (3.1)	
2	2 (3)	2 (5.3)	25 (10.6)	4 (16)	4 (8.3)	37 (8.9)	
3	34 (50.7)	16 (42.1)	101 (42.8)	14 (56)	22 (45.8)	187 (45.2)	
4	14 (20.9)	15 (39.5)	55 (23.3)	2 (8)	10 (20.8)	96 (23.2)	
5	15 (22.4)	5 (13.2)	42 (17.8)	1 (4)	11 (22.9)	74 (17.9)	
6			5 (2.1)			5 (1.2)	
MV	3.57	3.61	3.47	2.64	3.54	3.46	0.002

RBC: Rutherford Becker class; MV: mean value; n: number. Values are n (%). ANOVA was used.

**Table 6:** Uni- and multivariable logistic regressions for identifying risk factors for target lesion restenosis/reocclusion.

	Univariable Analysis			Multivariable Analysis		
	OR ^8^	95% CI ^9^	*p*-Value	Adj. ^10^ OR	95% CI	Adj. *p*-Value
**Smoking status ^1^**						
Current smoker	1.787	1.082; 2.982	0.024	1.831	1.05; 3.229	0.034
Former smoker	1.083	0.661; 1.781	0.752	1.339	0.779; 2.318	0.294
**Comorbidities ^2^**						
Diabetes mellitus	0.825	0.542; 1.26	0.372			
Renal disease	0.588	0.378; 0.917	0.019	0.663	0.407; 1.083	0.099
**Intervention details**						
Pre-interventional occlusion ^3^	5.103	3.28; 8.018	0.000	4.788	3.043; 7.609	0.000
Popliteal Intervention ^4^	1.758	1.163; 2.668	0.008	1.761	1.116; 2.792	0.015
Final vessel diameter	1.012	0.775; 1.333	0.930			
Total length of stent	1.002	1; 1.004	0.087			
**Stentlocation ^5^**						
Superficial femoral artery mid	0.778	0.285; 2.12	0.621			
Superficial femoral artery distal	0.667	0.278; 1.579	0.359			
Popliteal artery	1.756	0.814; 3.746	0.146			
Multisegmental	1.697	0.827; 3.417	0.142			
**Number of patent below the knee arteries ^6^**						
1	3.142	0.79; 13.311	0.101			
2	2.250	0.575; 9.391	0.240			
3	2.803	0.699; 11.965	0.142			
**Type of stent ^7^**						
Supera	0.910	0.413; 1.888	0.806			
Viabahn	1.290	0.414; 4.306	0.666			
BioMimics	0.730	0.301; 1.703	0.473			
Zilver	0.524	0.207; 1.277	0.161			

^1^ Reference: non-smoking; ^2^ reference: absence of specific comorbidity; ^3^ reference: pre-interventional stenosis; ^4^ reference: non-popliteal intervention; ^5^ reference: superficial artery proximal; ^6^ reference: zero-perfused-below-the-knee arteries; ^7^ reference: Smart stent; ^8^ odds ratio; ^9^ 95% confidence interval; ^10^ adjusted.

## Data Availability

No new data were created or analyzed in this study. Data sharing is not applicable to this article.

## References

[1] Bennett M.R., O’Sullivan M. (2001). Mechanisms of angioplasty and stent restenosis: Implications for design of rational therapy. Pharmacol. Ther..

[2] Minar E., Pokrajac B., Maca T., Ahmadi R., Fellner C., Mittlbock M., Seitz W., Wolfram R., Potter R. (2000). Endovascular brachytherapy for prophylaxis of restenosis after femoropopliteal angio-plasty: Results of a prospective randomized study. Circulation.

[3] Stonebridge P.A., Hoskins P.R., Allan P.L., Belch J.F. (1996). Spiral laminar flow in vivo. Clin. Sci..

[4] Berry J.L., Santamarina A., Moore J.J.E., Roychowdhury S., Routh W.D. (2000). Experimental and computational flow evaluation of coronary stents. Ann. Biomed. Eng..

[5] Chen Z., Fan Y., Deng X., Xu Z. (2009). Swirling flow can suppress flow disturbances in endovascular stents: A numerical study. ASAIO J..

[6] Piorkowski M. (2018). Stentimplantation: Selbstexpandierbare bioaktive Stentsysteme. Periphere Arterielle Interventionen.

[7] Stonebridge P.A., Brophy C.M. (1991). Spiral laminar flow in arteries?. Lancet.

[8] Tosaka A., Soga Y., Iida O., Ishihara T., Hirano K., Suzuki K., Yokoi H., Nanto S., Nobuyoshi M. (2012). Classification and Clinical Impact of Restenosis After Femoropopliteal Stenting. J. Am. Coll. Cardiol..

[9] Rutherford R.B., Becker G.J. (1991). Standards for evaluating and reporting the results of surgical and percutaneous therapy for peripheral arterial disease. J. Vasc. Interv. Radiol..

[10] Cheng C.P., Choi G., Herfkens R.J., Taylor C.A. (2010). The effect of aging on deformations of the superficial femoral artery due to hip and knee flexion: Potential clinical implications. J. Vasc. Interv. Radiol..

[11] https://www.veryanmed.com/international/products/biomimics-3d-vascular-stent-system/3d-helical-stent-design.

[12] Armstrong E.J., Bishu K. (2015). Supera self-expanding stents for endovascular treatment of femoropopliteal disease: A review of the clinical evidence. Vasc. Health Risk Manag..

[13] Caro C.G., Seneviratne A., Heraty K.B., Monaco C., Burke M.G., Krams R., Chang C.C., Coppola G., Gilson P. (2013). Intimal hyperplasia following implantation of helical-centreline and straight-centreline stents in common carotid arteries in healthy pigs: Influence of intraluminal flow ^†^. J. R. Soc. Interface.

[14] Shinke T., Robinson K., Burke M.G., Gilson P., Mullins L.P., O’Brien N., Heraty K.B., Taylor C., Cheshire N.J., Caro C.G. (2008). Novel Helical Stent Design Elicits Swirling Blood, Flow Pattern and Inhibits Neo-intima Formation in Porcine Carotid Arteries. Circulation.

[15] Zeller T., Gaines P.A., Ansel G.M., Caro C.G. (2016). Helical Centerline Stent Improves Patency: Two-Year Results From the Randomized Mimics Trial. Circ. Cardiovasc. Interv..

[16] Armstrong E.J., Singh S., Singh G.D., Yeo K., Ludder S., Westin G., Anderson D., Dawson D.L., Pevec W.C., Laird J.R. (2013). Angiographic characteristics of femoropopliteal in-stent restenosis: Association with long-term outcomes after endovascular intervention. Catheter. Cardiovasc. Interv..

[17] Ihnat D.M., Duong S.T., Taylor Z.C., Leon L.R., Mills J.L., Goshima K.R., Echeverri J.A., Arslan B. (2008). Contemporary outcomes after superficial femoral artery angioplasty and stenting: The influence of TASC classification and runoff score. J. Vasc. Surg..

[18] Shibuya T., Shintani T., Edogawa S., Satoh H. (2013). A review of surgically treated patients with obstruction after stenting in the femoropopliteal artery region. Ann. Vasc. Dis..

[19] Iida O., Nanto S., Uematsu M., Ikeoka K., Okamoto S., Nagata S. (2009). Influence of Stent Fracture on the Long-Term Patency in the Femoro-Popliteal Artery: Experience of 4 Years. JACC Cardiovasc. Interv..

[20] Diaz J.A., Villegas M., Tamashiro G., Micelli M.H., Enterrios D., Balestrini A., Tamashiro A. (2004). Flexions of the popliteal artery: Dynamic angiography. J. Invasive Cardiol..

[21] Rits J., van Herwaarden J., Jahrome A., Krievins D., Moll F. (2008). The incidence of arterial stent fractures with exclusion of coronary, aortic, and non-arterial settings. Eur. J. Vasc. Endovasc. Surg..

[22] Scheinert D., Scheinert S., Sax J., Piorkowski C., Bräunlich S., Ulrich M., Biamino G., Schmidt A. (2005). Prevalence and clinical impact of stent fractures after femoropopliteal stenting. J. Am. Coll. Cardiol..

[23] Lichtenberg M., Zeller T., Gaines P., Piorkowski M. (2022). BioMimics 3D vascular stent system for femoropopliteal interventions. Vasa.

[24] Hong S.-J., Ko Y.-G., Shin D.-H., Kim J.-S., Kim B.-K., Choi D., Hong M.-K., Jang Y. (2015). Outcomes of Spot Stenting Versus Long Stenting After Intentional Subintimal Approach for Long Chronic Total Occlusions of the Femoropopliteal Artery. JACC Cardiovasc. Interv..

[25] London N., Srinivasan R., Naylor A., Hartshorne T., Ratliff D., Bell P., Bolia A. (1994). Subintimal angioplasty of femoropopliteal artery occlusions: The long-term results. Eur. J. Vasc. Surg..

[26] Davies M.G., Saad W.E., Peden E.K., Mohiuddin I.T., Naoum J.J., Lumsden A.B. (2008). Percutaneous superficial femoral artery interventions for claudication--does runoff matter?. Ann. Vasc. Surg..

[27] Sullivan T.M., Zeller T., Nakamura M., Caro C.G., Lichtenberg M. (2018). Swirling Flow and Wall Shear: Evaluating the BioMimics 3D Helical Centerline Stent for the Femoropopliteal Segment. Int. J. Vasc. Med..

[28] Sullivan T.M., Zeller T., Nakamura M., Gaines P.A., on behalf of the MIMICS-2 Trial Investigators (2021). Treatment of Femoropopliteal Lesions With the BioMimics 3D Vascular Stent System: Two-Year Results From the MIMICS-2 Trial. J. Endovasc. Ther..

